# Prolonged production cycles in commercial laying hens and its bone repercussions

**DOI:** 10.1007/s11250-026-04879-0

**Published:** 2026-02-02

**Authors:** Denise C. Sousa, Ieverton C. C. Silva, Carlos B. V. Rabello, Lilian F. A. Souza, Webert A. Silva, Thaiza H. T. Fernandes, Ana P. M. Tenório, Valdemiro A. S. Júnior, Fabiano S. Costa

**Affiliations:** 1 Federal University of Piauí (UFPI/CPCE) and Graduate Program in Veterinary Medicine, Federal Rural University of Pernambuco (PPGMV-UFRPE), Recife, Brazil; 2 Focus Diagnóstico Veterinário and Maurício de Nassau Educational Center (UNINASSAU), Recife, Brazil; 3 Department of Animal Science, Federal Rural University of Pernambuco (UFRPE), Recife, Brazil; 4 Department of Veterinary Medicine (DMV), Federal Rural University of Pernambuco (UFRPE), Recife, PE Brazil

**Keywords:** Bone mineral density, Laying hens, Osteoporosis, Computed tomography, Skeletal health, Animal welfare

## Abstract

This study aimed to evaluate the impact of prolonged production cycles on bone quality in Dekalb White laying hens, focusing on bone mineral density (BMD), histopathological alterations, and serum biochemical parameters. A total of 10 Dekalb White hens at 83 weeks of age were assessed. Bone mineral density was evaluated using quantitative computed tomography (QCT). Histopathological analysis of the tibiae was performed, and serum biochemical markers related to bone metabolism were measured. The mean cortical tibial BMD was 847.19 ± 100.47 mg/cm³, with significant differences among tibial regions, as demonstrated by repeated measures ANOVA (*P = 0.003* for the right tibia and *P* = 0.002 for the left tibia), with higher values observed in the medial region compared with the proximal region. Histopathology revealed signs of osteoporosis, including increased cortical porosity, trabecular bone resorption, Haversian canal enlargement, and a higher number of active osteoclasts. No significant correlations were observed between BMD values and serum biochemical parameters (Pearson’s and Spearman’s correlations; *P* > 0.05). These findings indicate that extended laying cycles combined with cage confinement negatively impact skeletal health in aging laying hens. The integration of advanced imaging techniques such as QCT with histopathological evaluation proved effective for detecting bone fragility and early skeletal deterioration.

## Introduction

The genetic selection of commercial laying hens has markedly increased productivity, allowing production cycles to extend beyond 100 weeks. This intensification of egg production imposes substantial physiological demands, particularly on skeletal metabolism, due to sustained calcium mobilization throughout the laying cycle (Hanlon et al. 2022). Each eggshell requires approximately 10% of the hen’s total body calcium, maintaining the skeleton in a continuous state of remodeling and increasing the risk of structural bone depletion over time (Fathi et al. 2007; Iqbal et al. 2017; Lopez et al. 2018; Jones et al. 2025).

The performance and health of hens in the late production phase are increasingly studied to sustain profitable egg production, as birds become more vulnerable due to declining ovarian function and diminished ability to handle stress and disease. These physiological changes often lead to reduced laying rates, poorer egg quality, and more bone health issues (Almalamh et al. 2024; Arulnathan et al. 2024).

Bone fragility has therefore become one of the main constraints in modern egg production, contributing to mobility problems, reduced feed intake, and compromised welfare (Paludo 2016). During eggshell formation, calcium is primarily mobilized from medullary bone, but structural bone may also be affected, leading to cortical thinning and increased fracture risk (Whitehead, 2004). These issues are aggravated by confinement and intensive genetic selection (Whitehead and Fleming 2000).

The skeletal quality of laying hens can be evaluated through various methods, including traditional laboratory analyses such as bone ash content and the Seedor index (Seedor 1995), as well as bone breaking strength tests. Additionally, imaging techniques such as dual-energy X-ray absorptiometry (DEXA), micro-computed tomography (micro-CT), and quantitative computed tomography (QCT) enable the measurement of bone mineral density (BMD) and a detailed assessment of bone morphology (Korver et al., 2004; Chen and Kim 2020). However, imaging-based data for aged commercial hens remain limited, mainly due to small sample sizes and methodological constraints (Jones et al. 2025).

This study aimed to evaluate skeletal health in aged laying hens subjected to extended production cycles by integrating quantitative computed tomography, histopathological analysis, and serum biochemical profiling.

## Materials and methods

### Animals and experimental conditions

The hens were sourced from the Poultry Research Laboratory (LAPAVE) of the Department of Animal Science at the Federal Rural University of Pernambuco (Latitude: 8°01’11.3"S; Longitude: 34°57’14.6"W), located in Recife, Pernambuco, Brazil. A total of 10 Dekalb White laying hens, aged 83 weeks with an average body weight of 1.515 ± 0.03 kg, were randomly selected for this study. All experimental procedures were approved by the Animal Ethics Committee of the Federal Rural University of Pernambuco (CEUA/UFRPE, protocol no. 8680290224) and followed the guidelines of the Brazilian Federal Council of Veterinary Medicine (CFMV Resolution 1000/2012). The birds were housed in cages measuring 100 cm in length, 45 cm in width, and 40 cm in height, with a stocking density of 6 hens/m². Cages were equipped with trough feeders and automatic nipple drinkers. Water was provided ad libitum, and feed was offered once daily according to the nutritional requirements outlined in the breed manual, which was formulated based on each production stage (Rostagno et al. 2017). The diet composition is shown in Table 1.

**Table 1:** Composition of the diet fed to 83-week-old dekalb white laying hens

Ingredients	%	Chemical and Energy Composition	
Corn	61.65	Metabolizable energy (kcal/kg)	2.800
Soybean meal	23.38	Crude protein (%)	22.60
Soybean oil	1.80	Dry matter (%)	88.17
Fine Limestone	9.96	Ash (%)	13.11
Dicalcium phosphate	1.51	Sodium (%)	0.20
Sodium bicarbonate	0.15	Chloride (%)	0.20
Salt	0.30	Potassium (%)	0.60
DL-methionine (99.5%)	0.39	Phosphorus (%)	0.37
L-lysine (98.5%)	0.17	Calcium (%)	4.20
L-threonine (98.5%)	0.13		
Choline chloride (60%)	0.20		
Mineral premix¹	0.20		
Vitamin premix²	0.15		
Total	**100**		

¹ Mineral premix composition: Availa Mn, 80 g/kg of product; Availa Zn, 120 g/kg of product; Availa Cu, 100 g/kg of product; Availa Fe, 100 g/kg of product; Availa Se, 1,000 mg/kg of product; calcium iodate, 628 g/kg. ² Vitamin premix composition: vitamin A (12,000,000 IU/kg), vitamin D₃ (3,500,000 IU/kg), vitamin E (50,000 IU/kg), vitamin K₃ (3.0 g/kg), vitamin B₁ (2.5 g/kg), vitamin B₂ (6.5 g/kg), vitamin B₆ (5.0 g/kg), vitamin B₁₂ (30,000 µg/kg), niacin (40.0 g/kg), folic acid (1.0 g/kg), pantothenic acid (10.0 g/kg), biotin (0.20 g/kg)

A lighting program of 16 h per day was implemented, consisting of 12 h of natural light supplemented with 4 h of artificial light, following the Dekalb White Nutrition Guide (2009). Temperature and relative humidity were monitored daily using a datalogger (HOBO U12-012) placed at the ends of the poultry house and a digital thermohygrometer (Incoterm, model 7663.02.0.00) located centrally. Recorded values showed an average maximum temperature of 33.5 °C, minimum of 24.4 °C, and average relative humidity of 56%.

### Blood sampling and biochemical analysis

Blood samples were collected from the ulnar vein at approximately 9:00 a.m. using pediatric EDTA tubes for complete blood counts and plain tubes with clot activator for serum biochemical analyses. Parameters evaluated included ionized calcium, alkaline phosphatase (ALP), phosphorus, potassium, aspartate aminotransferase (AST), urea, and creatinine. Samples were temporarily stored in a thermal box with reusable ice packs for approximately 40 min before being transported to the laboratory and analyzed within 24 h after collection.

### Computed tomography (CT) scanning

Hens were anesthetized with a combination of midazolam hydrochloride (0.5 mg/kg) and ketamine hydrochloride (10 mg/kg), administered intramuscularly. CT scans were performed using a helical CT scanner (SIEMENS CT VA1). Imaging parameters included 1 mm slice thickness, bone and soft tissue reconstruction filters, 120 kV, automatic tube current (mA), and scan speed of one rotation per second. The hens were positioned in dorsal recumbency with a positioning cradle, and scans were obtained in a craniocaudal direction.

**Fig. 1:**
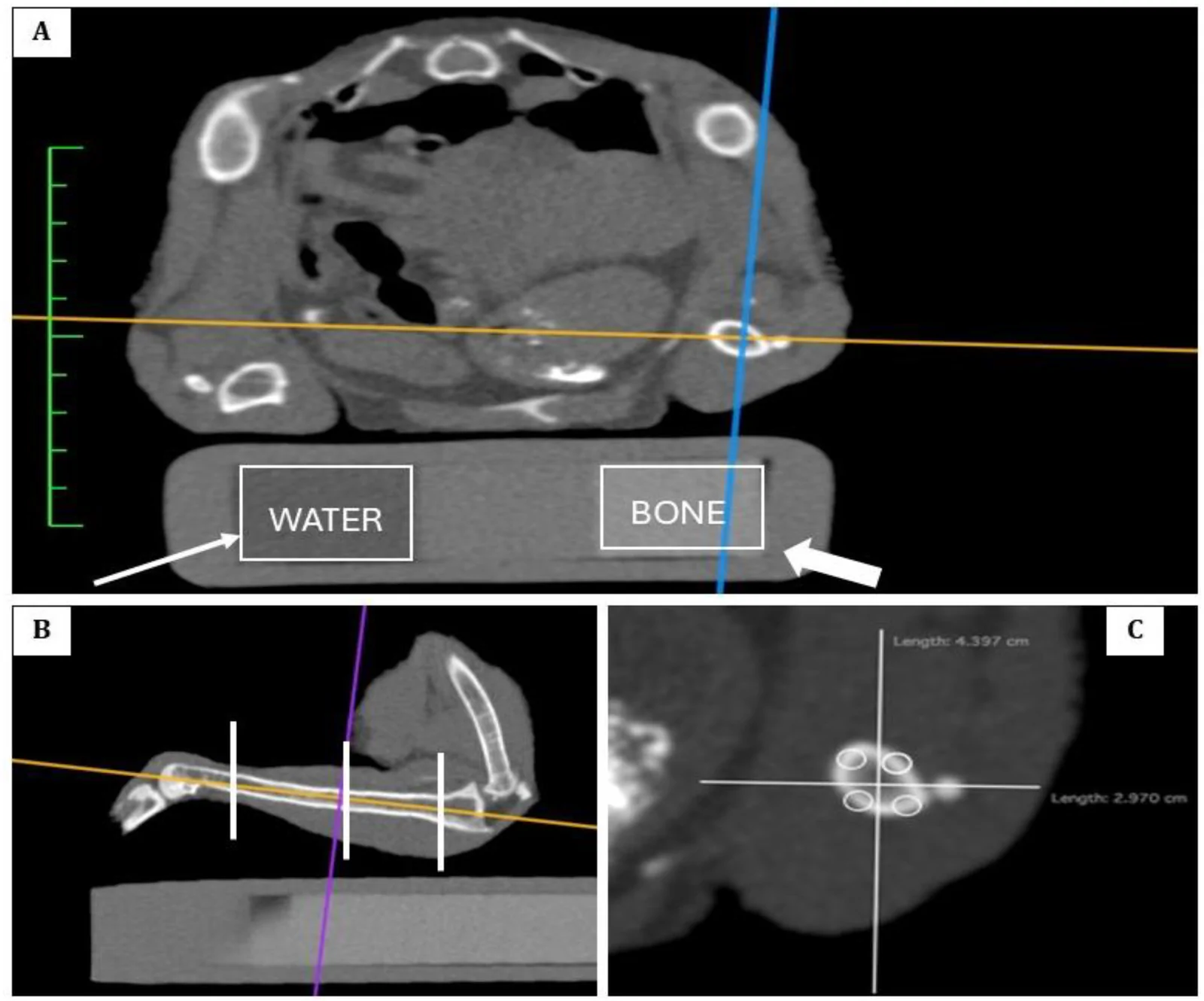
Computed tomography (CT), (**A**) calibration phantom containing calcium hydroxyapatite reference objects with densities of 0 (water) and 200 mg/cm³ (bone) used for calibration. (**B**) Sagittal CT view of the tibia showing the three diaphyseal levels (proximal, medial, and distal) where cortical bone radiodensity measurements were obtained. (**C**) Transverse CT section of the tibia with circular regions of interest (ROIs) positioned in four quadrants to measure bone density

A quantitative CT calibration phantom containing calcium hydroxyapatite reference objects with densities of 0 and 200 mg/cm³ was used for calibration (Fig. 1A).

### Histopathological sampling and processing

Hens were euthanized following the guidelines of Resolution 1000/2012 of the Brazilian Federal Council of Veterinary Medicine (CFMV, 2012). The tibiae were collected for histopathological analysis. Samples were decalcified in formic acid, fixed in 10% neutral buffered formalin for 48 h, and processed routinely for paraffin embedding. Sections were prepared and stained with hematoxylin and eosin according to Tolosa et al. (2003).

### Histological analysis

Histological assessments were performed qualitatively using a light microscope. The evaluation focused on structural composition and morphological alterations of bone tissue. Histological slides were digitized using a Leica DM500 microscope equipped with a scientific digital camera.

### CT image analysis

CT images were analyzed using Horos software (version 1.1.7, Horos, Purview, Annapolis, MD, USA) on a MacBook Air workstation. Radiodensity measurements of cortical bone were taken from transverse sections at three diaphyseal levels of the tibiae: proximal, medial, and distal (Fig. 1B). Each region was subdivided into four quadrants, and circular regions of interest (ROIs) were used to obtain bone density measurements (Fig. 1C). Radiodensity values were expressed in Hounsfield Units (HU) and corrected using phantom references for water (HUw) and bone (HUb). Bone mineral density in cm³ of hydroxyapatite (BMD) was calculated using the following formula: BMD = 200 × (HUt / (HUb – HUw**)**, where HUt represents the radiodensity of the measured bone (mg/cm³ of hydroxyapatite), HUb the radiodensity of the bone phantom (200 mg/cm³ hydroxyapatite), and HUw the radiodensity of the water phantom (0 mg/cm³ hydroxyapatite).

### Statistical analysis

Data are presented as mean ± standard deviation (SD). Data normality was verified using the Shapiro-Wilk test prior to applying parametric analyses. Comparisons between right and left tibiae, overall means, and between bone regions (proximal, medial, distal) were performed using repeated measures ANOVA (F-test) with Greenhouse–Geisser correction. Bonferroni post hoc tests were applied for pairwise comparisons when significant differences were detected.

Paired Student’s t-tests were used when the assumption of normal distribution was met. Pearson’s correlation was applied when both variables were normally distributed, and Spearman’s correlation was used otherwise. A significance level of 5% (*p* < 0.05) was adopted. All analyses were performed using IBM SPSS Statistics, version 25.

## Results

The serum biochemical profile of the hens is summarized in Table 2. Overall, ionized calcium, potassium, and AST showed low variability, suggesting stable metabolic status among individuals. In contrast, ALP, urea, and creatinine exhibited greater dispersion, indicating heterogeneous metabolic responses possibly related to age and the physiological stage of the birds. These findings are consistent with normal biochemical ranges for commercial laying hens and reflect the expected metabolic adjustments during prolonged egg production.

**Table 2:** Mean ± standard deviation (SD), median, 25th and 75th percentiles (P25 and P75), and reference values of serum biochemical parameters in 83-week-old dekalb white laying hens

Variable	Mean ± SD	Median (P25; P75)	Reference values
Phosphorus (mg/dL)	3.85 ± 1.39	3.61 (3.10; 4.85)	5 to 7*
Potassium (mmol/L)	3.79 ± 0.49	3.69 (3.52; 3.92)	2 to 4*
AST (U/L)	164.67 ± 20.29	160.23 (151.84; 173.87)	< 275*
Urea (mg/dL)	3.68 ± 3.36	2.42 (2.06; 3.53)	< 5*
Ionized calcium (mmol/L)	1.20 ± 0.19	1.19 (1.05; 1.28)	1.0 to 1.6*
Creatinine (mg/dL)	0.30 ± 0.21	0.22 (0.19; 0.35)	0.1 to 0.4*
ALP (U/L)	632.41 ± 432.07	511.60 (311.91; 908.02)	339–736**

*THRALL et al., 2015. **Garlich, J. D., 1974.AST: aspartate aminotransferase; ALT: alanine aminotransferase; ALP: alkaline phosphatase; mg/dL: milligrams per deciliter; mmol/L: millimoles per liter; U/L: international units per liter

The mean cortical bone mineral density (BMD) of the tibiae was 847.19 ± 100.47 mg/cm³ (Table 3). Repeated measures ANOVA with Greenhouse–Geisser correction revealed significant differences among tibial regions (*p* = 0.003 for the right and *p* = 0.002 for the left tibia). Bonferroni post hoc comparisons indicated that the medial region exhibited significantly higher BMD values than the proximal region, whereas differences between the medial and distal regions were not statistically significant.

**Table 3:** Mean ± standard deviation (SD), median, 25th and 75th percentiles (P25 and P75) of cortical bone mineral density in proximal, medial, and distal regions of the tibiae of 83-week-old dekalb white laying hens

Variable	Tibiae		*P*-value
	Right Tibia	Left Tibia	
	Mean ± SD	Mean ± SD	
	Median (P25; P75)	Median (P25; P75)	
Proximal ROI	799.29 ± 93.38 ^(B)^	798.04 ± 89.82 ^(B)^	p(1) = 0.925
	819.02 (731.32; 863.29)	794.97 (735.71; 867.35)	
Medial ROI	907.78 ± 113.97 ^(A)^	911.96 ± 122.28 ^(A)^	p(1) = 0.778
	965.30 (800.04; 995.64)	940.46 (819.44; 983.90)	
Distal ROI	833.46 ± 130.88 ^(AB)^	832.58 ± 110.87 ^(B)^	p(1) = 0.969
	830.96 (731.18; 917.24)	889.78 (743.72; 910.95)	
p-value	p^(2)^ = 0.003*	p^(2)^ = 0.002*	
Side mean	846.84 ± 102.63	847.53 ± 100.85	p(1) = 0.947
	889.10 (751.69; 917.21)	883.57 (775.86; 934.76)	
Overall mean	847.19 ± 100.47		
	888.82 (763.77; 925.94)		

ROI: region of interest

(*) Significant difference at 5%

(1) Paired Student’s t-test between sides

(2) Repeated measures ANOVA with Greenhouse–Geisser correction comparing regions by side, with Bonferroni post hoc

Note: Different letters in parentheses indicate significant differences between regions

No significant correlations were observed between serum biochemical markers and BMD (Pearson’s *r* = -0.12 to 0.25, *p* > 0.05; Table 4).

**Table 4:** Correlations between biochemical variables and cortical bone mineral density measurements of the tibiae of 83-week-old dekalb white laying hens

Variable	Tibiae *r* (*p*)
Phosphorus	0.30 (0.392) (1)
Potassium	0.16 (0.651) (2)
AST	–0.19 (0.603) (2)
Urea	0.02 (0.960) (2)
Ionized calcium	0.33 (0.355) (1)
Creatinine	0.14 (0.700) (2)
ALP	–0.47 (0.173) (1)

(*) Statistically significant at 5%

(1) Pearson correlation

(2) Spearman correlation

AST: aspartate aminotransferase; ALT: alanine aminotransferase; ALP: alkaline phosphatas

**Fig. 2:**
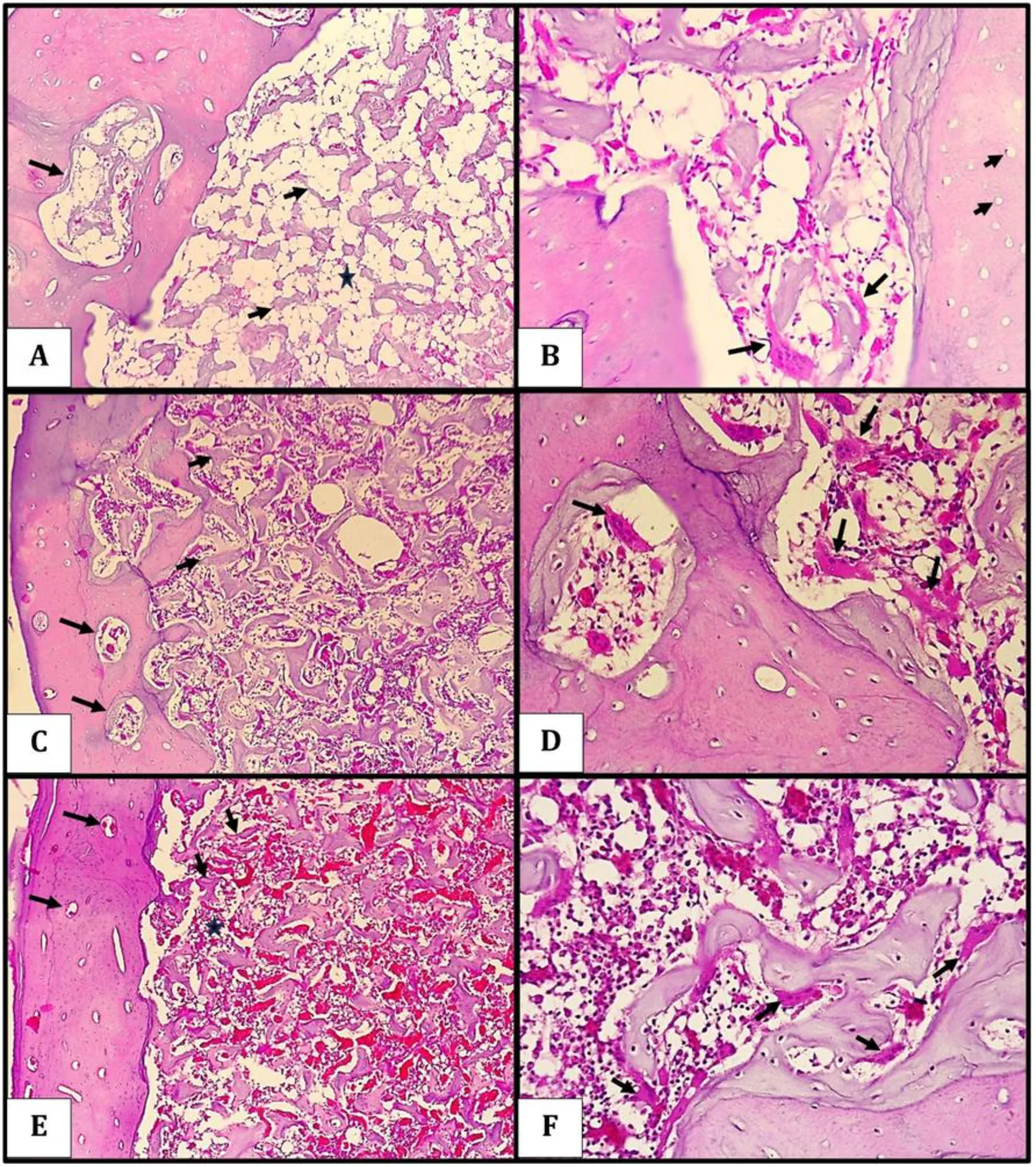
Histological analysis of decalcified tibial cortical bone stained with Hematoxylin and Eosin (H&E). (**A**) Enlargement of Haversian canals with fusion of two Haversian systems (arrows). Remnants of bone lamellae surrounded by inactive osteoblasts, blood vessels, and adipocytes are visible. In the medullary region, there is a reduction in trabecular bone (short arrow) and an increase in yellow bone marrow (unilocular adipose tissue – star). (**B**) Increased number of osteoclasts (arrows) and enlarged osteocyte lacunae indicating osteocytic osteolysis (short arrows) in the cortical bone. (**C**) Expansion of Haversian canals due to osteoclastic resorption (arrows). In the medullary region, there is reduced trabecular bone (short arrow) and increased lymphomyeloid marrow (star).(**D**) Activated osteoclasts performing bone resorption in the Haversian system (arrow) and adhered to trabecular bone surfaces in the medullary region (short arrow).(**E**) Slight enlargement of Haversian canals (arrows), reduction in trabecular bone (short arrow), and increased lymphomyeloid bone marrow (star).(**F**) Numerous activated osteoclasts attached to trabecular bone surfaces (short arrows), indicating intense bone resorption activity

Histological analysis of the tibial cortical bone revealed enlargement of Haversian canals, presence of bone lamellae remnants surrounded by inactive osteoblasts, blood vessels, and adipocytes. In the medullary region, there was a reduction in trabecular bone and an increase in yellow bone marrow. A marked increase in the number of osteoclasts was observed, along with evidence of osteoclastic resorption expanding the Haversian systems. Additionally, enlarged osteocyte lacunae and Haversian canal expansion were evident in the cortical bone due to osteoclastic remodeling. Numerous activated osteoclasts were observed attached to trabecular bone surfaces, actively engaged in bone resorption (Fig. 2).

## Discussion

Recent studies in the laying poultry industry have utilized quantitative computed tomography (QCT) to evaluate bone quality in response to dietary modifications, particularly those involving mineral supplementation (Medeiros-Ventura et al. 2023; Santos et al. 2024). Besides evaluating mineral density, QCT has proven effective for assessing bone structure and morphological changes (Milisits et al. 2013, 2015; Szentirmai et al. 2013; Donkó et al. 2018).

In the present study, QCT allowed the identification of site-specific differences in tibial density, which were later supported by histological findings. This integrative interpretation reinforces the applicability of advanced imaging tools in detecting early skeletal deterioration in aging hens.

With the growing emphasis on animal welfare, particularly in alternative production systems, employing advanced imaging methods becomes essential to support more effective management practices that enhance skeletal health in laying hens (Jones et al. 2025). Therefore, the integration of QCT with histopathological evaluation represents a powerful approach for detecting early indicators of bone fragility, especially in long-cycle commercial layers.

Following the imaging-based evaluation, it is crucial to interpret these variations within the context of bone morphology and biomechanical function. The tibia is a standard bone used in avian skeletal studies due to its rapid growth, load-bearing function, and strong correlation with calcium and phosphorus metabolism (Nunes et al. 2013; Toscano et al. 2013; Hanlon et al. 2022). Its high sensitivity to calcium imbalance makes it an effective parameter for evaluating bone mineral density (BMD) and overall skeletal integrity (Cloft et al. 2018).

Compared with the values reported by Santos et al. (2024), who found mean tibial BMDs of 703–760 mg/cm³ in White LSL-Lite hens at 98 weeks, the hens in the present study (847 mg/cm³ at 83 weeks) exhibited approximately 12–17% higher mineral density. This difference likely reflects the earlier sampling age and ongoing calcium absorption capacity. However, compared with younger hens supplemented with mineral complexes (Medeiros-Ventura et al. 2023; ~930–980 mg/cm³), the present BMD values were about 10–15% lower, consistent with the expected age-related decline in intestinal calcium absorption (Diana et al. 2021) and the progressive reliance on skeletal calcium mobilization (Gregory and Wilkins, 1989). These results suggest that even before the late laying phase, significant bone demineralization may already occur, reinforcing the need for early monitoring of bone health.

Conversely, the BMD values reported here were lower than those observed by Medeiros-Ventura et al. (2023), who found significantly higher bone density in Dekalb White hens at 68 weeks of age supplemented with amino acid–mineral complexes, with or without phytase. Similarly, Schreiweis et al. (2004) documented progressive BMD increases in Leghorn hens from 15 to 55 weeks, reinforcing that the reproductive phase has a direct influence on skeletal health. Taken together, these comparisons highlight how age and nutrient bioavailability interact to modulate mineral deposition and bone resistance throughout the productive lifespan.

In this study, BMD distribution along the tibia was heterogeneous, with higher values in the medial and distal regions. This pattern aligns with findings from Santos et al. (2024) and Medeiros-Ventura et al. (2023). Barreiro et al. (2009) suggested that the higher mineral density in these regions reflects biomechanical adaptation to the forces applied by muscles and ligaments during growth and body mass gain, thereby stimulating localized mineralization. Such regional variation is consistent with the functional anatomy of the tibia, where stress-induced remodeling contributes to maintaining structural resilience.

A statistically significant difference in BMD was observed between the proximal and medial tibial regions (*p* = 0.003). Regmi et al. (2016) explained that this variation may be due to site-specific skeletal responses and the anisotropic nature of bone. Natural bone curvatures and muscle attachment sites influence structural composition, resulting in localized skeletal adaptations (Regmi et al. 2017). These biomechanical dynamics, when associated with metabolic calcium turnover, illustrate the complexity of maintaining skeletal integrity during prolonged egg production cycles.

Despite calcium’s well-established role in maintaining BMD, no significant correlation was found between BMD values obtained via QCT and serum ionized calcium levels in this study. This differs from Neijat et al. (2011), who reported strong correlations between these parameters. The lack of correlation here may be associated with the timing of blood collection. Sloan et al. (1974) demonstrated that serum calcium levels in hens follow a circadian rhythm, peaking around 10:00 PM, which is considered the optimal time for assessing ionized calcium levels. Thus, temporal variation in calcium homeostasis likely contributed to the absence of direct correlations, highlighting the importance of sampling standardization in future bone metabolism studies.

Histological analysis revealed clear signs of osteoporosis. Osteoporosis in laying hens is characterized by progressive bone mass loss during the laying cycle, leading to fragile bones prone to fractures, especially in caged systems (Whitehead and Fleming 2000). Nutritional deficiencies, particularly of calcium and phosphorus, are major contributors to reduced BMD and increased fracture risk (Jiang et al. 2019; Zhao et al. 2020; Teng et al. 2020). Certain supplementation strategies, such as using coarse calcium particles, can enhance bone strength (Fleming et al. 2006). These findings align with the current histological observations of increased porosity and osteoclastic resorption, which are typical markers of high bone turnover in aged layers.

Additionally, lipid metabolism disorders associated with dietary imbalances can affect bone quality (Jiang et al. 2013). Aging increases cortical porosity, decreases cortical volume, enhances osteoclastic activity, and promotes Haversian canal expansion (Yamada et al. 2021). Elevated fibroblast growth factor 23 (FGF23) expression in older hens further disrupts calcium and phosphorus homeostasis, exacerbating osteoporosis (Gloux et al. 2020).

Genetic factors also play a critical role in bone strength. Selective breeding for stronger bones has been shown to reduce fracture incidence significantly (Koning et al. 2020; Bishop et al. 2000; Whitehead and Fleming 2000), sometimes having a greater impact than nutritional interventions (Fleming et al. 2006). While hens raised in cage-free systems typically exhibit better bone quality than caged hens, the risk of fractures does not disappear entirely (Fleming et al. 2006; Whitehead and Fleming 2000). This reinforces that environmental enrichment and genetic selection should be viewed as complementary rather than alternative approaches to improving skeletal health in poultry.

These factors contextualize the findings of this study, considering that the hens evaluated were Dekalb White, a strain characterized by prolonged productive cycles and high egg output. Their confinement in cages and advanced age (83 weeks) likely contributed to the histopathological changes observed. These included bone mass reduction, increased porosity, and accelerated bone remodeling, indicated by elevated numbers of osteoclasts and osteoblasts — hallmarks of high bone turnover (Yamada et al. 2021; Zhao et al. 2020; Jiang et al. 2019).

Finally, by integrating QCT-derived BMD data with histological and biochemical findings, this study provides a holistic understanding of how prolonged egg production impacts skeletal metabolism.

Recent research underscores the importance of understanding the regulatory mechanisms of calcium and phosphorus metabolism in aging laying hens. The decline in intestinal calcium absorption with age, coupled with increased reliance on skeletal mobilization to sustain egg production, exacerbates osteoporosis and fracture risk (Diana et al. 2021; Sinclair-Black et al. 2023). This physiological scenario aligns with the skeletal profile of the hens in this study.

## Conclusion

This study evaluated bone quality in laying hens subjected to prolonged production cycles, revealing significant reductions in bone mineral density and signs of osteoporosis. The combined use of QCT and histopathology was effective in identifying cortical porosity, trabecular thinning, and increased osteoclastic activity. Advanced age, high metabolic demand, and confinement were found to exacerbate skeletal fragility, particularly in caged systems. Although the number of birds evaluated was limited and data collection occurred at a single time point, the study reinforces the importance of early monitoring and nutritional, genetic, and welfare strategies to prevent demineralization and promote sustainability in egg production.

## Data Availability

Data will be made available on reasonable request.’
